# Quality of life in young and middle age adult temporomandibular disorders patients and asymptomatic subjects: a systematic review and meta-analysis

**DOI:** 10.1186/s12955-021-01727-7

**Published:** 2021-03-10

**Authors:** Lucas Bozzetti Pigozzi, Duziene Denardini Pereira, Marcos Pascoal Pattussi, Carmen Moret-Tatay, Tatiana Quarti Irigaray, João Batista Blessmann Weber, Patrícia Krieger Grossi, Márcio Lima Grossi

**Affiliations:** 1 School of Health and Life Sciences, Pontifical Catholic University of Rio Grande Do Sul (PUCRS), Avenida Ipiranga 6681 Prédio 6, Building 11, 9th Floor, Porto Alegre, RS 90619-900 Brazil; 2 Public Health, Vale Do Rio Dos Sinos University (UNISINOS), Av. Unisinos, 950 - Cristo Rei, São Leopoldo, RS 93020-190 Brazil; 3 Faculty of Psychology, Universidad Católica de Valencia San Vicente Mártir, Avenida de La Ilustración 4, 46100 Burjassot, Valencia Spain; 4 Dipartimento Di Neuroscienze Salute Mentale E Organi Di Senso (NESMOS), Università Sapienza Di Roma, Rome, Italy; 5 School of Humanities, Pontifical Catholic University of Rio Grande Do Sul (PUCRS), Avenida Ipiranga 6681 Prédio 9, Porto Alegre, RS 90619-900 Brazil

**Keywords:** Systematic review, Meta-analysis, Quality of life, Temporomandibular joint disorders, Epidemiology

## Abstract

**Aims:**

To compare the difference in the quality of life between temporomandibular disorders (TMD) patients and non-TMD subjects diagnosed with the Research Diagnostic Criteria for Temporomandibular Disorders (RDC/TMD) or the Diagnostic Criteria for Temporomandibular Disorders (DC/TMD).

**Methods:**

Medical Literature Analysis and Retrieval System Online (MEDLINE), Excerpta Medica database (EMBASE) and Latin American and Caribbean Health Sciences Literature (LILACS) databases were searched in studies published in English and Portuguese. The search was performed by two independent reviewers in duplicate. A manual search and the gray literature were also included. The inclusion criteria were clinical studies that used the RDC/TMD axis I and quality of life with standard questionnaires in young and middle-aged adult population (18–55 years). The data were analyzed quantitatively by combining the results in a meta-analysis using forest plots. The measure of effect used was the standardized mean difference (SMD) in depression levels. The Newcastle–Ottawa Scale (NOS) was used to evaluate the quality of the studies. The publication bias was assessed by funnel plots. The initial search included 806 articles without duplications.

**Results:**

Twenty-four articles were included in the final systematic review. Of these, 9 were included in the meta-analysis, where it was shown a statistically significant in all axis I groups: (a) global TMD—groups I, II and III combined, N = 3829, SMD (95% CI) = 1.06 (0.65–1.51), p = 0.000; (b) group I—muscle disorders, N = 3,056, SMD (95% CI) = 0.82 (0.45–1.18), p = 0.000; (c) group II—disc displacements, N = 3,184, SMD (95% CI) = 0.59 (0.26–0.91), p = 0.000; and (d) group III—arthralgia/arthritis/arthrosis, N = 2781, SMD (95% CI) = 0.98 (0.59–1.36), p = 0.000. When compared to controls.

**Conclusions:**

Quality of life is affected in all axis I TMD patients, especially in groups I and III with higher pain intensity and disability as compared to group II.

## Introduction

According to the World Health Organization (WHO) (1995) [1], quality of life is defined as the individual's perception of his/her position in life in the context of culture and according to the value systems of the society in which you live, and in relation to your goals, expectations, standards and concerns. In the past, health and quality of life were directly associated with the medical model. At present, the socio-environmental model guides many aspects of health strategies and investments, bringing the body and mind as two directly interconnected units. Therefore, we cannot consider the oral cavity as an environment isolated from the body that will interfere alone in the quality of life of the individual; and because of that, questionnaires about quality of life should evaluate the individual as a whole, in different dimensions for each questionnaire [2].

The development of this type of research that relates the quality of life to the most diverse diseases found in the population is of great importance due to the allocation of public and private financial resources to the most emergency and impactful situations, based on the equity of care principle. In addition, studying the quality of life influences the clinical decision-making process and the practices that aim at greater personal, social and work income [3].

A recent number of studies have associated quality of life with temporomandibular disorders (TMD). However, these studies have a variable methodology and diverse results, both regarding the quality of life and the diagnosis of TMD [4–6]. A literature review reported that TMD patients have worse quality of life than non-TMD subjects, but without reporting specific data to the TMD diagnostic groups according to the Research Diagnostic Criteria for Temporomandibular Disorders (RDC/TMD) axis I (i.e., muscle disorders or group I, disc displacements or group II, and arthralgia/arthritis/arthrosis or group III), and without performing a meta-analysis [7]. Therefore, a systematic review with meta-analysis from TMD studies which used the RDC/TMD as a diagnostic tool describing the axis I diagnoses is still missing. This is paramount in order to verify if different TMD diagnostic groups have different levels of quality of life.

The primary aim of this study was to perform a systematic review and meta-analysis of the perception of the quality of life in TMD patients as compared to non-TMD subjects in clinical and population studies that have used both the RDC/TMD, and the Diagnostic Criteria for Temporomandibular Disorders (DC/TMD), and standard quality of life questionnaires. The secondary objectives were to compare the quality of life in both TMD patients and non-TMD subjects diagnosed by the RDC/TMD axis I diagnostic groups: (a) muscle disorders or group I, (b) disc displacements or group II, and (c) arthralgia/arthritis/arthrosis or group III.

## Methods

### Study design and ethical approval

A systematic review was performed with meta-analysis following the Preferred Reporting Items for Systematic Reviews and Meta-Analyses (PRISMA) statement guidelines [8]. The research protocol was registered in the International Prospective Register of Systematic Reviews (PROSPERO) database (2017: CRD42017072229). The project was approved by the Research Ethics Committee of the School of Health and Life Sciences, Pontifical Catholic University of Rio Grande do Sul (PUCRS) (2017: SIPESQ # 8244), Brazil.

### Research question

This study followed the research question guidelines of the PRISMA Statement [8]. The PICOT strategy was followed, where "P" refers to the target population or problem of interest, "I" to the intervention under investigation, "C" to the control group, "O" to the outcome, and "T" to the types of studies included in the review [8]. The research question was: "Is there a difference in the quality of life in the population that presents TMD compared to the population without TMD?” In this context, "P" were young and middle-aged adult patients, "I" refers to temporomandibular disorders, "C" refers to the absence of temporomandibular disorders, "O" refers to the quality of life, and "T" refers to cross-sectional, cohort and case–control studies.

### Inclusion and exclusion criteria

The inclusion criteria were: (a) to be an observational study (i.e. cross-sectional, cohort, or case–control studies) in young and middle-aged adult subjects (18–55 years of age), (b) to use the RDC/TMD or the DC/TMD as the diagnostic criteria for temporomandibular disorders, and (c) to use standard questionnaires that measure the quality of life. The studies must have been published since 1992, and they must have been published in the Portuguese and English languages.

The exclusion criteria involved: (a) studies in which the disease or outcome was not TMD (e.g., headaches, neuropathic, facial pain, lip and cleft palate patients, etc.), (b) studies in which the patients underwent previous TMD treatments (i.e., orthodontics, oral surgery, oral splints, medication, etc.), (c) studies in patients with a history of facial trauma or rheumatic diseases, and d) studies which did not use standard research diagnostic questionnaires for TMD and/or quality of life diagnoses.

### Search strategy

General search terms with the controlled descriptors for each database were used, employing the Biochemistry Health Sciences Descriptors (DeCS), the Medical Subject Heading (MeSH) from MEDLINE terms, and the descriptors and terms published in the literature. These terms and descriptors are found in Table 1. To connect these terms, we used the Boolean terms "AND" and "OR" in order to expand and restrict the search spectrum. The databases used were Medical Literature Analysis and Retrieval System Online (MEDLINE), Excerpta Medica database (EMBASE) and Latin American and Caribbean Health Sciences Literature (LILACS). For the gray literature, the Networked Digital Library of Theses and Dissertations/Global Electronic Theses and Dissertations Service (NDLTD/Global EDT Search), *Coordenação de Aperfeiçoamento de Pessoal do Ensino Superior* Brazilian Government Bank of Theses and Dissertations (CAPES/BDTD), Open Gray, and Google Scholar databases were consulted. In addition, a manual search was also performed. The total electronic search of all databases was performed in between August and December 2019.

**Table 1:** All databases searched, search terms used, and number of articles found per database

Database searched	Search strategy and terms	Articles retrieved
Pubmed/medline^*^	("Temporomandibular Joint Disorders"[Mesh] or “Temporomandibular joint disorder” or “Disorder, Temporomandibular Joint” or “Disorders, Temporomandibular Joint” or “Joint Disorder, Temporomandibular” or “Joint Disorders, Temporomandibular” or “Temporomandibular Joint Disorder” or “TMJ Disorders” or “Disorder, TMJ” or “Disorders, TMJ” or “TMJ Disorder” or “Temporomandibular Disorders” or “Disorder, Temporomandibular” or “Disorders, Temporomandibular” or “Temporomandibular Disorder” or “Temporomandibular Joint Diseases” or “Disease, Temporomandibular Joint” or “Diseases, Temporomandibular Joint” or “Joint Disease, Temporomandibular” or “Joint Diseases, Temporomandibular” or “Temporomandibular Joint Disease” or “TMJ Diseases” or “Disease, TMJ” or “Diseases, TMJ” or “TMJ Disease” or “Temporomandibular joint dysfunction syndrome” or “Temporomandibular joint pain” or “Temporomandibular pain” or “Craniomandibular Disorders” or “Craniomandibular Disorder” or “Disorder, Craniomandibular” or “Disorders, Craniomandibular” or “Craniomandibular Diseases” or “Craniomandibular Disease” or “Disease, Craniomandibular” or “Diseases, Craniomandibular” or “Chronic orofacial pain” or “Orofacial Pain” or “Craniofacial pain” or “Chronic craniofacial pain”) AND ("Quality of Life"[Mesh] or “Quality of life” or “Quality of lives” or “Life Quality” or “Health-Related Quality Of Life” or “Health Related Quality Of Life” or “Life Style” or “Karnofsky Performance Status” or “Sickness Impact Profile” or “Value of Life” or “Oral Health-related Quality of life” or “Oral Health Impact Profile” or “World Health Organization Quality of Life” or “Social Dental Indicators” or “General Oral Health Assessment Index” or “General quality of life” or “The Dental Impact Profile” or “Subjective Oral Health-Related Quality of Life Measure” or “The Dental Impact Daily Living” or “Oral Impacts on Daily Performances”)	491
EMBASE^**†**^	(“Temporomandibular joint disorder” or “Disorder, Temporomandibular Joint” or “Disorders, Temporomandibular Joint” or “Joint Disorder, Temporomandibular” or “Joint Disorders, Temporomandibular” or “Temporomandibular Joint Disorder” or “TMJ Disorders” or “Disorder, TMJ” or “Disorders, TMJ” or “TMJ Disorder” or “Temporomandibular Disorders” or “Disorder, Temporomandibular” or “Disorders, Temporomandibular” or “Temporomandibular Disorder” or “Temporomandibular Joint Diseases” or “Disease, Temporomandibular Joint” or “Diseases, Temporomandibular Joint” or “Joint Disease, Temporomandibular” or “Joint Diseases, Temporomandibular” or “Temporomandibular Joint Disease” or “TMJ Diseases” or “Disease, TMJ” or “Diseases, TMJ” or “TMJ Disease” or “Temporomandibular joint dysfunction syndrome” or “Temporomandibular joint pain” or “Temporomandibular pain” or “Craniomandibular Disorders” or “Craniomandibular Disorder” or “Disorder, Craniomandibular” or “Disorders, Craniomandibular” or “Craniomandibular Diseases” or “Craniomandibular Disease” or “Disease, Craniomandibular” or “Diseases, Craniomandibular” or “Chronic orofacial pain” or “Orofacial Pain” or “Craniofacial pain” or “Chronic craniofacial pain”) AND (“Quality of life” or “Quality of lives” or “Life Quality” or “Health-Related Quality Of Life” or “Health Related Quality Of Life” or “Life Style” or “Karnofsky Performance Status” or “Sickness Impact Profile” or “Value of Life” or “Oral Health-related Quality of life” or “Oral Health Impact Profile” or “World Health Organization Quality of Life” or “Social Dental Indicators” or “General Oral Health Assessment Index” or “General quality of life” or “The Dental Impact Profile” or “Subjective Oral Health-Related Quality of Life Measure” or “The Dental Impact Daily Living” or “Oral Impacts on Daily Performances”)	564
LILACS^**‡**^	(“Temporomandibular joint disorder(s)” or “Craniomandibular disorder(s)” or “Temporomandibular joint dysfunction syndrome” or “Temporomandibulares disorder(s)” or “Temporomandibular disorders” or “Temporomandibular joint” or “Temporomandibular joint pain” or “Chronic orofacial pain” or “Orofacial pain” or “Craniofacial pain” or “Chronic craniofacial pain”) AND ("Quality of Life" or “Quality of life” or “Quality of lives” or “Life Quality” or “Health-Related Quality Of Life” or “Health Related Quality Of Life” or “Life Style” or “Karnofsky Performance Status” or “Sickness Impact Profile” or “Value of Life” or “Oral Health-related Quality of life” or “Oral Health Impact Profile” or “World Health Organization Quality of Life” or “Social Dental Indicators” or “General Oral Health Assessment Index” or “General quality of life” or “The Dental Impact Profile” or “Subjetive Oral Helth-Related Quality of Life Measure” or “The Dental Impact Daily Living” or “Oral Impacts on Daily Performances”)	128

* Medical Literature Analysis and Retrieval System Online (MEDLINE)

^†^ Excerpta Medica database (EMBASE)

^‡^ Latin American and Caribbean Health Sciences Literature (LILACS)

### Study selection and extraction of data

**Fig. 1:**
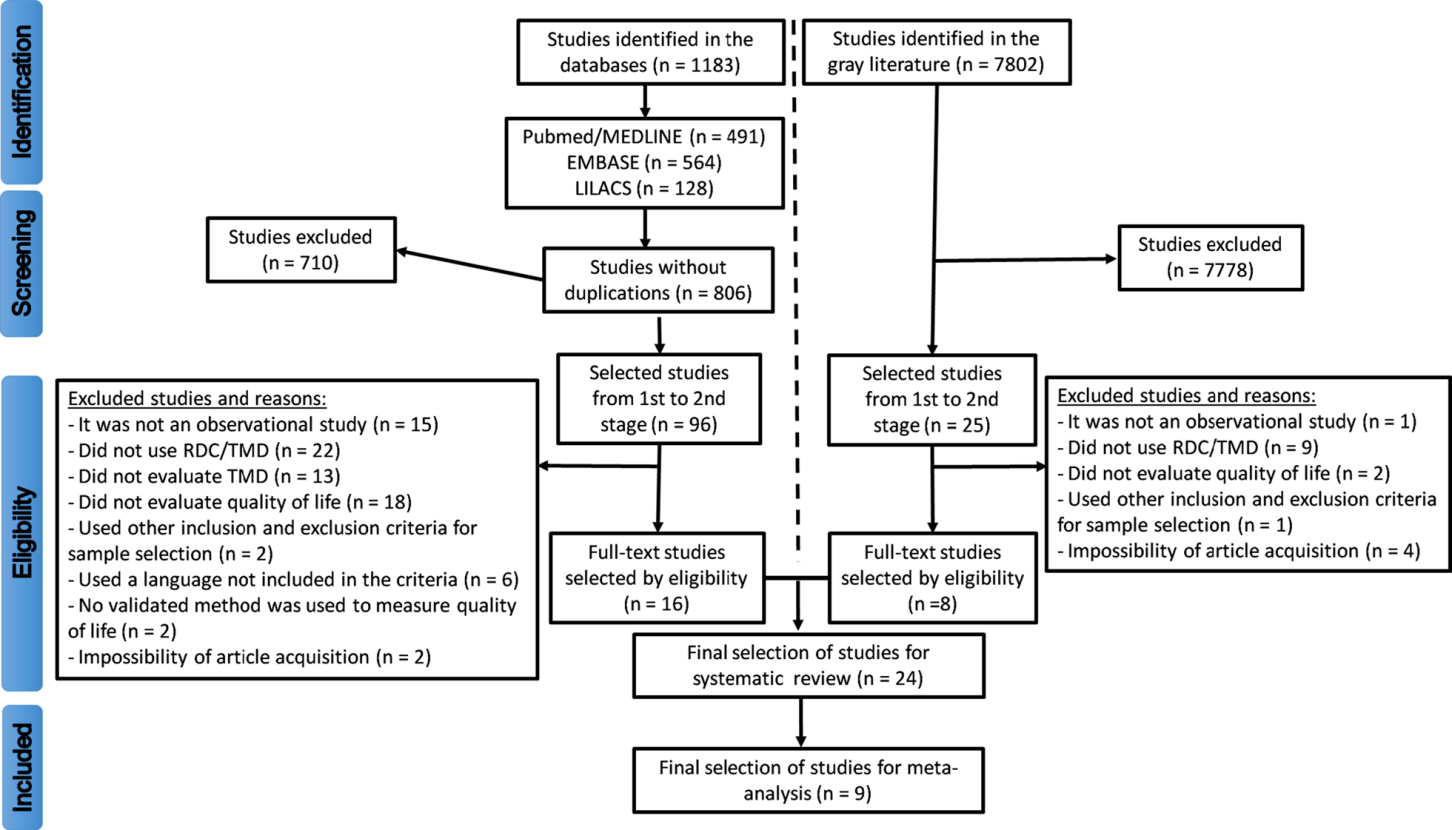
Flowchart for identification and selection of studies

The Endnote Web program (Thomson Reuters®, New York, USA) was used to create the database, where the articles could be selected and organized. The articles selection was performed in two stages by two independent reviewers: (a) first, reading the titles and abstracts and article selection by at least one of the reviewers individually, and (b) second, reading the full-text and article selection by both reviewers in agreement; when disagreement was present, a third reviewer was consulted. The articles that were eliminated at this stage for not meeting the inclusion criteria had the reason for their exclusion described in Fig. 1. For articles that were not available in full text in the database, or if the data in the article was incomplete or missing, an attempt was made to contact the author, and if not successful, the article was purchased.

For data extraction, an Excel worksheet was created (Microsoft Office®, Microsoft, Redmond, USA) based on the recommendations of the “Cochrane Handbook for Systematic Reviews” and the “Strobe initiative: guidelines on reporting observational studies”, also by two independent reviewers [9, 10]. Based on these recommendations, data extraction included the following information: (a) general information about the study (i.e., title, year and period of publication, first author, and country of origin); (b) information about the methodology (i.e., duration/follow-up of the study, study site, study design, diagnostic criteria for TMD and quality and life); (c) information about the sample (i.e., sample selection and collection method, sample size, age and gender distribution); (d) information about the outcome (i.e., the prevalence of outcome and comparison to controls); and (e) additional information (i.e., statistical methods involved, such as odds ratio or standard error).

### Data analysis and quality evaluation

The quality evaluation of the included articles was performed by the Newcastle–Ottawa Scale (NOS). This questionnaire is based on a star system used to classify observational studies, that is, a star is assigned for each quality of the specific item, providing a quick and direct view with a maximum score of seven stars. In NOS system, there are three main topics: (a) the first related to the selection of the groups, (b) the second related to the comparability between the groups, and (c) the third related to the verification of the exposure/outcome of interest [11].

In relation to the data, they were analyzed in a quantitative way by combining the results in a meta-analysis by presenting forest plot charts. Only observational studies that presented sufficient data for analysis (i.e., sample size, mean and standard deviation in both TMD and control groups) were included. A total of four meta-analyses were made: the first for global TMD (i.e., combined RDC/TMD axis I groups I, II, and III), and the others for groups I, II and III of the RDC/TMD axis I classification separately. Observational studies without a control group, articles that were not divided by groups of TMD, and articles that did not present data clearly were excluded from the meta-analysis.

The heterogeneity was analyzed by the I^2^ inconsistency test that assigns values from 0 to 100%, where 0 to 25% is considered low, 25 to 75% is considered intermediate, and over 75% is considered high [12]. As it was not possible to perform meta-analysis in all articles included in this review, the data were also analyzed qualitatively and presented in tables. The measure of effect used was the standardized mean difference (SMD) [13]. Publication bias was evaluated by means of funnel plots. The statistical program used was the STATA version 12 (StataCorp® LLC, USA, 2011).

## Results

### Systematic review

The data extraction from articles selected according to the inclusion and exclusion criteria were performed after the complete article reading. The data were inserted in a MicroSoft Excel Spreadsheet (Microsoft Office®) according to the guidelines of the “Cochrane Manual for Systematic Reviews” and the “Strobe initiative: guidelines on reporting observational studies” [9, 10].

Both the search strategy and the number of articles retrieved can be observed in Fig. 1. Table 2 presented a summary of the 24 articles selected, as well as their characteristics obtained in the data collection. Of all studies, 17 were case–control and seven were cross-sectional prevalence studies (i.e., without a control group); the oldest was from 2005. The RDC/TMD axis I was applied in all 24 studies, while axis II in only seven. Only eight case–control studies applied the RDC/TMD in both cases and controls, and the remaining only in cases. The DC/TMD axis I was used in one study for cases. Regarding the questionnaires measuring quality of life, 10 studies used the Oral Health Impact Profile (OHIP)—14, 5 used the OHIP—49, 1 used the OHIP but it did not report the version, 3 studies used the World Health Organization Quality of Life (WHOQOL)—BREF, and 5 used the Short Form-36 (SF-36).

**Table 2:** Description of observational studies which used the Research Diagnostic Criteria for Temporomandibular Disorders (RDC/TMD) to assess temporomandibular disorders (TMD) and a standard measure for quality of life

Author (year)	Title	Journal	Country	Diagnostic criteria used	Measurement of Qual. of life	Method of data collection	Number and qualification of the examiner	Case selection method	Control selection method
Ahn et al [18]	Objective and subjective assessment of masticatory function in patients with temporomandibular disorder in Korea	J Oral Rehabil	South Korea	RDC/TMD (did not report axes) in cases and controls	OHIP (no version informed)	Interview, CE andRx	A single specialist examiner	Patients seeking treatment at the dental clinic	Dental Students
Almoznino et al. [19]	Oral Health Related Quality of Life in Temporomandibular disorder patients	J Oral Facial Pain and Headache	Israel	DC/TMD and RDC/TMDaxis I in cases	OHIP-14	Interview, CE	Not informed	Patients seeking treatmentat the dental clinic	Patients from the integrated clinic without TMD
Bayat et al [6]	Oral health-related quality of life in patients with temporomandibular disorders: A case–control study considering psychological aspects	Int J Dent Hygiene	Iran	RDC/TMD axes I and II in cases	OHIP-14	Interview, CE	A single calibrated specialist examiner	Patients seeking treatmentat the dental clinic	Returning patients for maintenance
Karacayli et al [14]	The effects of chronic pain on oral healthrelated quality of life in patients with anterior disc displacement with reduction	Community Dental Health	Turkey	RDC/TMD (did not report axes) in cases	OHIP-14	Interview, CE and MRI	A single calibrated specialist examiner	Patients seeking treatmentat the dental clinic	Random sample from healthy patients
Miettinen. Lahti, Sipilä [15]	Psychosocial aspects of temporomandibular disorders and oral health-related quality-of-life	Acta Odontol Scand	Finland	RDC/TMD axis I in cases	OHIP-14	Interview, CE	Not informed	Patients seeking treatmentat the dental clinic	University students without TMD
Schierz et al [28]	Comparison of perceived oral health in patients with temporomandibular disorders and dental anxiety using oral health-related quality of life profiles	Qual Life Res	Germany	RDC/TMD (does not report axes) in cases	OHIP-14	Interview, CE	Severalexperiencedexaminers	Patients seeking treatmentat the dental clinic	Probabilistic sample from the general population
John et al [20]	Oral health-related quality of life in patients with temporomandibular disorders	J Orofac Pain	Germany	RDC/TMD axes I and II in cases	OHIP-49	Interview, CE	Small number of experienced dentists	Patients seeking treatment at the dental clinic	Probabilistic sample from the general population
Moufti et al [29]	The Oral Health Impact Profile: ranking of items for temporomandibular disorders	Europe J Oral Sci	Ukraine	RDC/TMD axes I and II in cases	OHIP-49	Interview, CE	Not informed	Patients from the health care system	Accompanying other patients who did not have TMD
Reissmann et al [21]	Functional and psychosocial impact related to specific temporomandibular disorder diagnoses	J Dent	Germany	RDC/TMD axes I and II in cases and controls	OHIP-49	Interview, CE	Small group of specialists	Patients seeking treatmentat the dental clinic	Probabilistic sample from the general population evaluated by the Helkimo-index without TMD
Rener-Sitar et al [22]	Oral health related quality of life in Slovenian patients with craniomandibular disorders	Coll Antropol	Slovenia	RDC/TMD axis I in cases and controls	OHIP-49	Interview, CE	Not informed	Patients seeking treatmentat the dental clinic	Randomized sample fromthegeneral urban population
Rener-Sitar et al [23]	Factors related to oral health related quality of life in TMD patients	Coll Antropol	Slovenia	RDC/TMD axis I in cases and controls	OHIP-49	Interview, CE	Not informed	Patients seeking treatmentat the dental clinic	Randomized sample from the general urban population
Barros et al [24]	The impact of orofacial pain on the quality of life of patients with temporomandibular disorder	J Orofac Pain	Brazil	RDC/TMD axis I	OHIP-14 (modified)	Interview, CE	Three calibrated examiners (did not report qualification)	Patients seeking treatmentat the dental clinic	Not informed
Blanco-Aguilera et al [16]	Application of an oral health-related quality of life questionnaire in primary care patients with orofacial pain and temporomandibular disorders	Med Oral Patol Cir Bucal	Spain	RDC/TMD (did not report axes)	OHIP-14	Interview, CE	A single calibrated specialist examiner	Sample of the population of the Public Health System	Not informed
Su et al [4]	Correlation between oral health-related quality of life and clinical dysfunction index in patients with temporomandibular joint osteoarthritis	J Oral Sci	China	RDC/TMD axis I	OHIP-14	Interview, CE	A single calibrated specialist examiner	Patients seeking treatmentat the dental clinic	Not informed
Tjakkes et al [25]	TMD pain: The effect on health related quality of life and the influence of pain duration	Health Qual Life Outcomes	Netherlands	RDC/TMD axes I and II	SF-36	Interview, CE	Not informed	Patients seeking treatmentat the dental clinic	Not informed
Resende et al [5]	Quality of life and general health in patients with temporomandibular disorders	Braz Oral Res	Brazil	RDC/TMD axes I and II	WHOQOL-Brev	Interview, CE	Not informed	Patients seeking treatmentat the dental clinic	Not informed
Portella, Smith, Guimarães [31]	Qualidade de vida em pacientes com disfunção temporomandibular: avaliação através do questionário SF-36	CAPES BDTD	Brazil	RDC/TMD axis I in cases	SF-36	Interview, CE	Many dentists	Patients seeking treatmentat the dental clinic	Patients seeking treatmentat the dental clinic
Trize, Marta [32]	Disfunção temporomandibular e sua associação com qualidade de vida	CAPES BDTD	Brazil	RDC/TMD axis I in cases and controls	SF-36	Interview, CE	A single calibrated examiner (did not inform qualification)	Patients seeking treatmentat the dental clinic	Patients seeking treatmentat the dental clinic
Castanharo, Junior [34]	Estudo da qualidade de vida em pacientes com disfunção temporomandibular e cefaleias primárias	CAPES BDTD	Brazil	RDC/TMD axis I in cases and controls	SF-36	Interview, CE	Several trained and calibrated researchers (did not report qualification)	Patients seeking treatmentat the dental clinic	Patients seeking treatmentat the dental clinic
Gui et al [33]	Quality of life in temporomandibular disorders patientes with localized and widespread pain	Braz J Oral Sci	Brazil	RDC/TMD axis I in cases	SF-36	Interview, CE	Calibrated examiners (did not report numbers)	Not informed	Not informed
Pigozzi et al [26]	Prevalence of quality of life in patients with temporomandibular disorders in an adult population-based case–control study in Southern-Brazil	Int J Prosthodont	Brazil	RDC/TMD axes I and II in cases and controls	WHOQOL-Brev	Interview, CE	A single calibrated and PhD student examiner	Randomized, representative sample from the population	Randomized, representative sample from the population
Da Silva, Barbosa [35]	Relação entre aspectos sociodemográficos, ansiedade e qualidade de vida com disfunção temporomandibular	CAPES BDTD	Brazil	RDC/TMD axis I in the cases and controls	WHOQOL-Brev	Interview, CE	A single calibrated examiner (did not report qualification)	Notinformed	Not informed
Lucena, Da Costa, De Góes [17]	O impacto da disfunção temporromandibular na qualidade de vida relacionada à saúde bucal	CAPES BDTD	Brazil	RDC/TMD axis I and II	OHIP-14	Interview, CE	A single calibrated and PhD student examiner	Patients seeking treatment at the dental clinic	Not informed
Rodrigues, Mazzatto [27]	Impacto da dor e do ruído articular na qualidade e no custo de vida de indivíduos com disfunção temporomandibular	CAPES BDTD	Brazil	RDC/TMD axis I	OHIP-14	Interview, CE	A single examiner (did not report qualification)	Patients referred from the Public Health System	Not informed

CE = clinical examination, RX = X-ray examination, MRI = Magnetic Resonance Imaging, CAPES BDTD = *Coordenação de Aperfeiçoamento de Pessoal de Nível Superior* Bank of Theses and Dissertations, WHOQOL = World Health Organization Quality of Life, SF-36 = Short Form 36, OHIP = Oral Health Impact Profile

Based on Table 3 results, the mean age of all participants in the included studies ranged from 20.93 to 50.93, confirming that the data from all included articles were from young and middle-aged adult patients from both genders [19, 33]. It was possible to observe that all selected studies indicated worse quality of life in TMD patients. Some studies have shown a direct relationship between both the duration of pain and pain intensity with poor quality of life in patients with TMD, showing that pain caused by TMD is one of the main reasons for the quality of life scores [6, 14–17]. Coherently, most articles clearly indicated that groups I and III, which have worse pain intensity, have worse quality of life as compared to group II of the RDC/TMD axis I [5, 18–27].

**Table 3:** Outcomes of the selected studies: prevalence (%) of temporomandibular disorders (TMD) by gender using the Research Diagnostic Criteria for Temporomandibular Disorders (RDC/TMD) by diagnostic group individually and by all diagnostic groups combined and itsrespectiveresults/conclusions

Author (year)	Measurement Quality of life	n (n woman)/ mean age (SD)	Total number of controls (n woman)/mean age (SD)	Results / conclusions
Ahn et al [18]	OHIP (does not report version)	51(32)/26.2(8.8)	20(5)/26.5(9.1)	OHIP scores were worse in the TMD group than in controls. The pain group presented the domains of physical pain (2.05), physical disability (2.15) and psychological incapacity (1.81), greater than the control group. There was no significant difference with MAI, but there was a higher correlation with FIA than with VAS. The FIA showed correlation with the 5 domains of OHIP, mainly physical incapacity and pain
Almoznino et al. [19]	OHIP-14	187(111)/21.12(3.83)	200(90)/20.93(3.74)	In TMD group, there were statistical differences for the following OHIP domains as compared to controls: physical pain, physical incapacity, psychological discomfort, and psychological incapacity. The groups with the worst results were: muscular and articular pain, followed by muscular only and articular only groups, but with no statistical difference between the last two groups. There was no difference in relation to the sociodemographic profile. There was an inverse relationship between pain and quality of life, mainly due to limitation of mouth opening, forced opening of the mouth, pain during opening, and limitation of lateral movements
Bayat et al [6]	OHIP-14	75(64)/34.3(12.3)	75(55)/29.1(6.1)	The TMD group had a statistically worse quality of life than controls, positively correlated with TMD severity, mainly related to duration of pain and the GCPS scale. There was no statistical difference regarding ageand gender in relation toquality of life prevalence, but severity was higher in women.The prevalence and severity of OHIP was 6 and 2 times higher respectively in the TMD group, and the factor that influenced the most was the psychological incapacity
Karacayli et al [14]	OHIP-14	37(23)/29(**)	37(23)/30(**)	In the OHIP, patients with disc displacement had worse quality of life than the control group, mainly inbothworse pain in the last 6 months and average intensity of pain in the last 6 months. In addition, a worse OHIP-14 score was observed in patients who had problems with smiling/laughing, teeth/face cleaning, swallowing, and talking. OHIP was significantly worse when pain intensity was also higher
Miettinen. Lahti, Sipilä [15]	OHIP-14	79(61)/43.5(13.1)	70(47)/25.3(6.5)	OHIP was worse in all RDC/TMD groups relative to controls, and it was also directly related to pain intensity. Women had an OHIP worse than men in all sub-items and also in relation to severity. OHIP was 3 times worse in the TMD than in the non-TMD group. Psychosocial factors were associated with TMD and impaired quality of life
Schierz et al [28]	OHIP-14	416(329)/37.4(16.2)	2026(1054)/43.3(16.2)	Patients with TMD had a statistically worse OHIP scores than both patients with anxiety and the general population, the last with the best quality of life
John et al [20]	OHIP-49	416(329)/37.4(16.2)	2026(1054)/43.3(16.2)	For OHIP, on the RDC/TMD axis I, there was better quality of life in patients with disc displacement without reduction as compared to the other two groups. However, they were statistically worse than the control group. Women had worse scores, but with no statistical difference. Regarding axis II, mandibular dysfunction had worse OHIP scores. There was greater somatization in the TMD group, with worse OHIP scores, as opposed to depression. However, both were higher than the general population
Moufti et al [29]	OHIP-49	110(92)/39(**)	110 (92)/38(**)	The study demonstrated statistical differences between patients with and without TMD in OHIP scores. The impact of pain and physical disability was substantial. The study also appeared to show a worse outcome on the impact of the overall oral health in quality of life among TMD patients, with worse scores reported in all items
Reissmann et al [21]	OHIP-49	471(358)/38.6(15.6)	35(16)/36.1(10.7)	The population with TMD had significantly worse OHIP scores than controls. Within the TMD groups, the worst OHIP score was for myofascial pain without limited opening, and the best OHIP score was for disc displacement group with reduction. Patients with DD without reduction had a significantlyworse OHIP scores than with reduction. Within group III, there was no significant statistical difference among arthralgia, arthritis and arthrosis. In the 3 TMD groups, group II had the best OHIP scores, differing statistically from groupsI (the worst) and group III. Groups I and III did not differ between themselves
Rener-Sitar et al [22]	OHIP-49	68(58)/36.54(13.76)	400(270)/41.38(12.66)	OHIP scores wereworse in the TMD population than in the controls. The best OHIP scores were in disc displacement with reduction, and the worst were in disc displacement without reduction with limited opening. There was no significant difference between genders
Rener-Sitar et al [23]	OHIP-49	81(65)/36.1(13.4)	400(291)/41.38(12.66)	Similar results were reported in relation to the previous study by the same authors; however, the worst OHIP scores were found inboth osteoarthritis and disc displacement without reduction with limited opening
Barros et al [24]	OHIP-14 (modif.)	83(69)/36.5(13.5)	–	Women presented worse impact in the functional limitation; in the other domains, there was no significant difference. There was statistical difference between groups I and III, but not against group II; and group III had the worst result. The severity of TMD was directly related to poorer quality of life
Blanco-Aguilera et al [16]	OHIP-14	407(364)/**♀**42.15(14.66) and **♂**41.48(17.28)	–	Women had a worse OHIP scores than men. OHIP still showed a significant and positive association between patients with both high intensity of pain without disability and poor perception of quality of life in relation to oral health. They also presented higher OHIP values for physical pain and psychological discomfort. The duration of pain over 1 year also interfered in OHIP by 6.5 points in relation to the group with less pain duration. Age and marital status were not significant
Su et al [4]	OHIP-14	541(407)/38.59(15.52)	–	Muscle sensitivity during palpation was related to worse OHIP scores in all domains. An increase in TMJ pain scores on palpation in HDI was significantly associated with worse OHIP total score and domains, with the exception of functional limitation
Tjakkes et al [25]	SF-36	95(90)/40.3(13.1)	–	There was statistical difference for SF-36 in RDC/TMD groups I and III in the following areas: physical functionality and pain in the body. But there was no significant difference between groups II and III. The other scores did not differ statistically amonggroups. Regarding TMD duration, patients with less than 1 year with diagnosed TMD presented better scores in physical functionality. However, those who had TMD for more than 1 years had an impact mainly on social commitment
Resende et al [5]	WHOQOL-Bref	43(43)/36.48(**)	–	The WHOQOL was worse for group II in the social aspect for the disc displacement with reduction. In the physical aspect, there was a significant association with all TMD groups, and it was directly related to pain severity. The worst WHOQOL scores were in the group with associated muscular and articular dysfunction
Portella, Smith, Guimarães [31]	SF-36	45(45)/32(10)	58(58)/33(10)	The TMD group presented SF-36 scores significantly worse than those in the control group in the following domains: functional capacity, physical appearance, pain, general health status, vitality, social aspects, emotional aspects and mental health
Trize, Marta [32]	SF-36	51(*)/**	51(*)/**	The TMD group showed worse quality of life than the group without TMD, in all absolute values, but it was statistically significant only for pain and mental health
Castanharo, Junior [34]	SF-36	228(200)/**	34(19)/**	There was a statistical difference for all domains between general TMD and controls. Regarding pain, the control group differed from the other threeRDC/TMD axis I groups. The TMD + headache group differed from both the TMD groupandthe headache group alone. For mental health, emotional and social aspect, and general health, the TMD + headache group had significantly worse scores than both the control group and headache group alone
Gui et al [33]	SF-36	76(76)/**	40(40)/50.93(12.34)	Patients in the TMD group with diffuse pain differed significantly in all components as compared to controls. In the TMD with localized pain, the emotional factor did not differ among subgroups. The domains of general health, mental, physical and psychological function did not differ between TMD with localized pain and controls
Pigozzi et al [26]	WHOQOL-Bref	584(*)/**	1048(*)/**	There was a significantly worse quality of life in all domains in both RDC/TMD axis I and II versus controls. Group I (muscle disorders), group III (arthralgia) and group III (osteoarthritis) had statistically significant difference in all domains as compared to controls. For group II (disc displacement), this difference was not observed in any domain. For group III with osteoarthrosis, there was no significant difference for the psychological, social and environmental domains, butonly for the physical domain. Pain intensity/severity was related to lower quality of life scores
Da Silva, Barbosa [35]	WHOQOL-Bref	60(*)/**	60(*)/**	In all domains, subjects without TMD showedsignificantlybetterquality of life and compared to TMD patients. In the WHOQOL-General, the subjects without TMD showed also significantbetterscores of quality of life. There were 9.2 times more chances of individuals with low quality of life of having TMD than those with medium to high quality of life scores
Lucena, Da Costa, De Góes [17]	OHIP-14	155(138)/37.3(12.9)	–	Pain interfered negatively in the quality of life, with greater impairment in the performance of the daily activities related to the physical domain, followed by the psychological and, with less impact, in the social activities. Psychological factors, such as depression, somatization, psychosocial incapacity, and pain intensity were significantly associated with quality of life impairment
Rodrigues, Mazzatto [27]	OHIP-14	80(70)/32.71(**)	–	TMD interfered in the quality of life in all three RDC/TMD axis I groups. Disc displacement with muscle pain had the worstquality of life, while the best was only for disc displacement. The severity of pain was also directly related to the worst quality of life scores

*It does not separate by case–control, they only report the total number of women in the study. ** It is not clear in the article. MAI—Mixing Ability Index

FIA—Food Intake Ability. VAS—Visual Analogue Scale. GCPS—Graded Chronic Pain Scale. HDI—Helkimo Clinical Dysfunction Index

WHOQOL = World Health Organization Quality of Life, SF-36 = Short Form 36, OHIP = Oral Health Impact Profile

### Meta-analysis

**Fig. 2:**
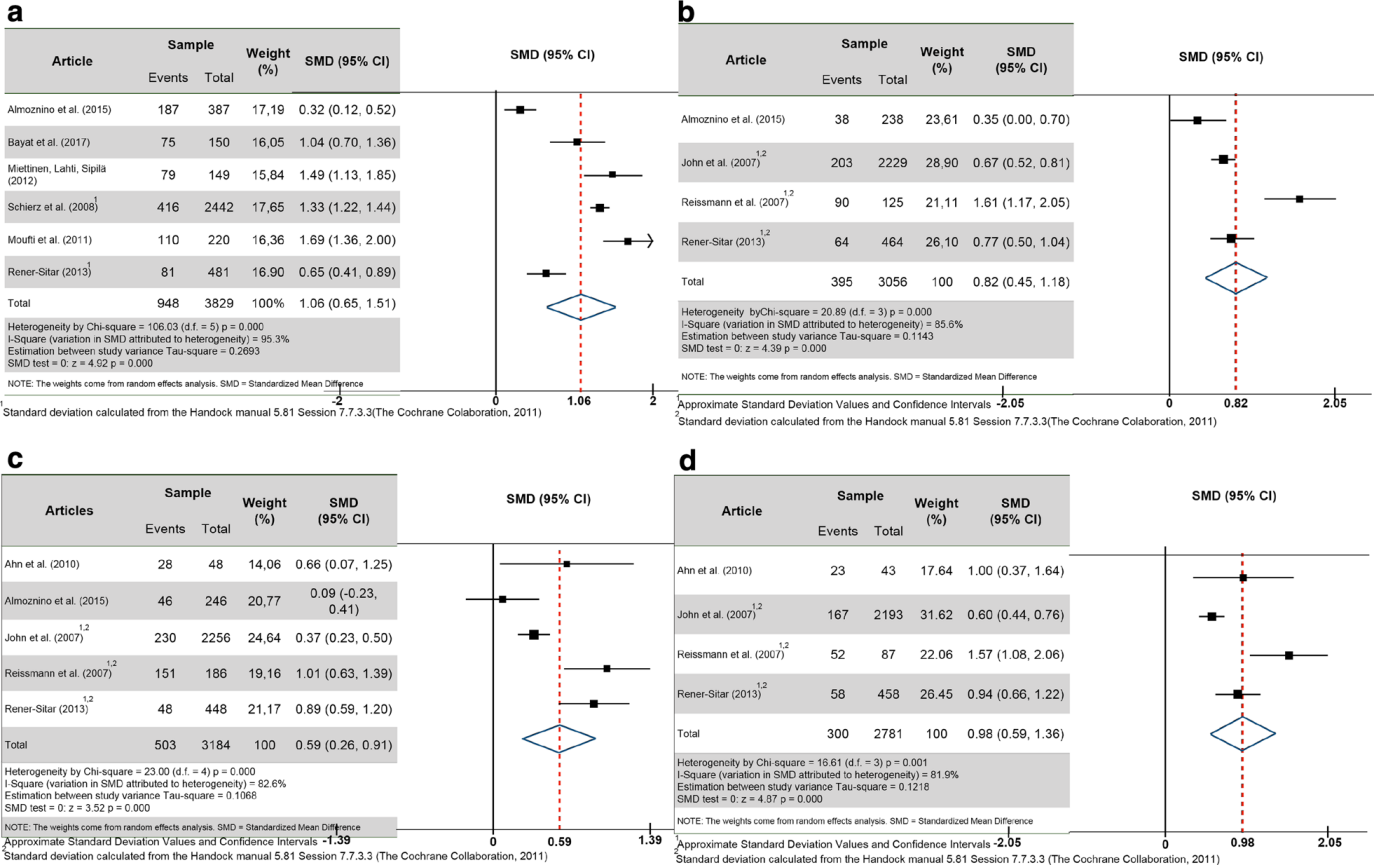
Forest plot of the differences of the standardized means. **a** Forest plot of the differences of the standardized means for TMD in a global aspect (RDC/TMD Axis I: groups I, II and III combined) versus controls without TMD diagnosis. **b** Forest plot of the differences of the standardized means for muscle disorders (RDC/TMD, Axis I, group I) versus controls without TMD diagnosis. **c** Forest plot of standardized mean differences for disc displacements (RDC/TMD, Axis I, group II) versus controls without TMD diagnosis. **d** Forest plot of the differences of the standardized means for arthralgia/arthritis/arthrosis (RDC/TMD, Axis I, group III) versus controls without TMD diagnosis

Due to the great variability in the included studies, only 9 studies were used in the meta-analysis (Fig. 2); because they were the only ones that clearly included the number of participants, the mean, the standard deviation per group, and compared TMD patients with the controls without TMD [6, 15, 18–21, 23, 28, 29]. Out of 24 studies, only 2 had a sample from a non-clinical origin for the TMD cases (i.e., population studies); therefore, most studies were from clinical populations seeking TMD care [16, 26]. However, included studies in the systematic review which had no control group were excluded from the meta-analysis [4, 5, 16, 17, 24, 25, 27, 30]. One article separated the RDC/TMD axis I groups into sub-groups (i.e., Ia, Ib, IIa, IIb, IIc, IIIa, IIIb, IIIc), and it was not possible to have accurate data for groups I, II and III [22]. Some studies used the SF-36 criteria for quality of life without presenting a general index, only its 7 sub-topics [31–33]. One study presented a SF-36 general data, but it could not be included in the meta-analysis due to the different methodology used [34]. Finally, one excluded study used the WHOQOL-BREV, but it did not report the patients’ origin [35].

Figure 2a included the six articles that reported the quality of life in global TMD patients (combined RDC/TMD axis I groups I + II + III) compared to those without TMD diagnosis; where the first four used the OHIP-14, and the last two used the OHIP-49. From the meta-analysis presented, it was possible to observe that there was a very high statistical difference in the quality of life between patients with and without TMD: (a) total sample = 3,829 subjects, (b) SMD (95% confidence interval) = 1.06 (0.65, 1.51), (c) heterogeneity I^2^ = 95.3%, and (d) Z test = 4.92, p = 0.000. Figure 2b refers to RDC/TMD axis I group I (i.e., muscle disorders) compared to patients without TMD diagnosis. In this analysis, it was only possible to include four articles, where one used the OHIP-14 and three used the OHIP-49. A very high statistical significance was also found: (a) total sample = 3,056 subjects, (b) SMD (95% confidence interval) = 0.82 (0.45, 1.18), (c) heterogeneity I^2^ = 85.6%%, and (d) Z test = 4.39, p = 0.000. Figure 2c compared the RDC/TMD group II (i.e., disc displacements) versus controls without TMD. Only five articles were included, where two used the OHIP-14 and three used the OHIP-49; and again, there was a highly statistically significant difference between the two groups: (a) total sample = 3,184 subjects, (b) SMD (95% confidence interval) = 0.59 (0.26, 0.91), (c) heterogeneity I^2^ = 82.6%, and (d) Z test = 3.52, p = 0.000. Figure 2d showed the RDC/TMD axis I group III (arthralgia/arthritis/arthrosis) versus controls without TMD. In this analysis, four articles were included, one used the OHIP-14 and three used OHIP-49; and once more a very highly statistically significant difference was found: (a) total sample = 2781 subjects, (b) SMD (95% confidence interval) = 0.98 (0.59, 1.36), (c) heterogeneity I^2^ = 81.9%, and (d) Z test = 4.87, p = 0.000.

Therefore, the results showed that in all RDC/TMD axis I groups, TMD patients have much worse quality of life as compared to non-TMD subjects. However, in the comparison among Fig. 2b–d; it was possible to observe a higher SMD in TMD patients from group III with 0.98, followed by groups I with 0.82, and II with 0.59. Therefore, RDC/TMD axis I groups with higher pain levels (i.e., groups I and III) had worse quality of life as compared to the one with lower pain levels (i.e., group II).

### Publication bias

**Fig. 3:**
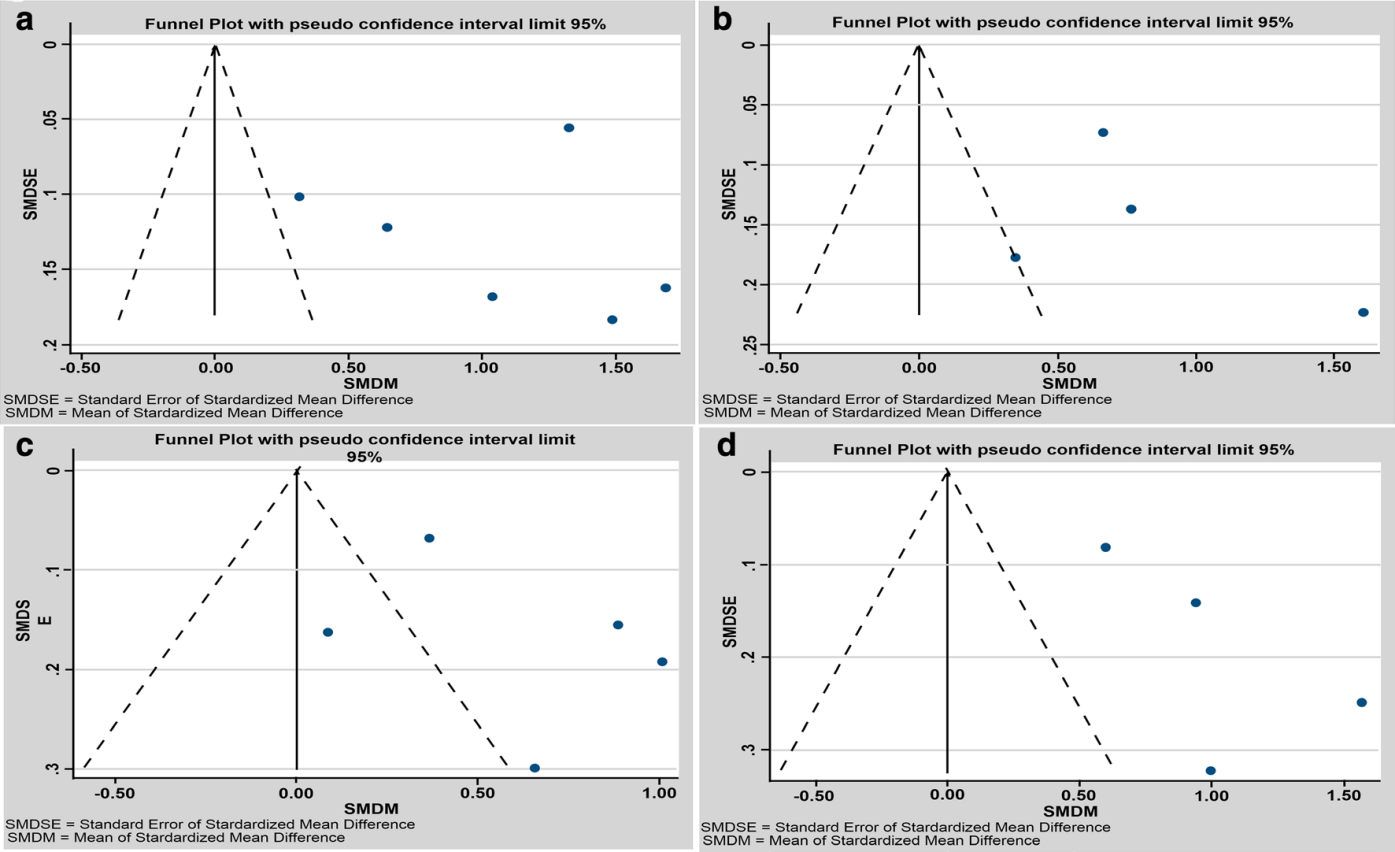
Funnel Plot of the differences of the standardized means. **a** Funnel Plot of the differences of the standardized means for TMD in a global aspect, (RDC/TMD Axis I: groups I, II and III) versus controls without TMD diagnosis. **b** Funnel Plot of the differences of the standardized means for muscle disorders (RDC/TMD, Axis I, group I) versus controls without TMD diagnosis. **c** Funnel Plot of the differences of the standardized means for disc displacements (RDC/TMD, Axis I, group II) versus controls without TMD diagnosis. **d** Funnel Plot of the differences of the standardized means for arthralgia/arthritis/arthrosis (RDC/TMD, Axis I, group III) versus controls without TMD diagnosis

We analyzed the publication bias of the studies included in the meta-analysis presented in Fig. 3. They were also divided according to RDC/TMD axis I: (a) global TMD (Fig. 3a), (b) group I (Fig. 3b), (c) group II (Fig. 3c), and (d) group III (Fig. 3d). The included studies were the same included in the meta-analysis for each respective group. The four graphs showed that all studies were on the right side of the funnel plots, indicating that patients with TMD have worse quality of life, presenting a publication bias towards positive results.

### Quality of the studies

Table 4 analyzed the quality of the 16 published articles only included in the NOS that was searched in the electronic databases. We can observe that the great majority of the studies presented a good methodological quality, but many presented biases in the outcome measurement method, without making it clear how the data collection was performed. In addition, a large part of the studies did not perform the RDC/TMD diagnosis in the control group, presenting the possibility of undiagnosed TMD patients among controls. This might reduce the actual difference between the test and control groups in the quality of life assessment.

**Table 4:** Result of the quality evaluation (Newcastle–Ottawa Scale—NOS star system) of the sixteen selected studies which were included in the final electronic selection database search

Study	Selection	Comparability	Outcome		Total
Ahn et al [18]	**★** 1C, 2C, 3A	**★★**	**★** 1D, 2A		4**★**
Almoznino et al [19]	**★★** 1C, 2A, 3A	**★★**	**★** 1D, 2A		5**★**
Bayat et al [6]	**★★** 1C, 2A, 3A	**★★**	**★** 1D, 2A		5**★**
Karacayli et al [14]	**★★** 1C, 2A, 3A	**★★**	**★** 1D, 2A		5**★**
Miettinen. Lahti, Sipilä [15]	**★** 1C, 2B, 3A	**★★**	**★** 1D, 2A		4**★**
Schierz et al [28]	**★★★** 1B, 2A, 3A	**★★**	**★** 1D, 2A		6**★**
John et al [20]	**★★★** 1B, 2A, 3A	**★★**	**★** 1D, 2A		5**★**
Moufti et al [29]	**★★** 1C, 2A, 3A	**★**	**★** 1D, 2A		4**★**
Reissmann et al [21]	**★★★** 1B, 2A, 3A	**★★**	**★** 1D, 2A		6**★**
Rener-Sitar et al [22]	**★★** 1C, 2A, 3A	**★★**	**★** 1D, 2A		4**★**
Rener-Sitar et al [23]	**★★** 1C, 2A, 3A	**★★**	**★** 1D, 2A		4**★**
Barros et al [24]	**★** 1C, 2A, 3B	0	**★** 1D, 2A		2**★**
Blanco-Aguilera et al [16]	**★★★** 1B, 2A, 3B	0	**★** 1D, 2A		4**★**
Su et al [4]	**★** 1C, 2B, 3A	**★★**	**★** 1D, 2A		4**★**
Tjakkes et al [25]	**★** 1C, 2B, 3A	0	**★** 1D, 2A		2**★**
Resende et al [5]	**★** 1C, 2B, 3A	0	**★** 1D, 2A		2**★**

## Discussion

This study aimed to compare the perception of quality of life in TMD patients and non-TMD subjects in clinical studies that have used both the RDC/TMD, the DC/TMD and valid quality of life questionnaires. Based on the findings in this systematic review (Tables 2 and 3), it was possible to observe that all included studies showed some relationship between the presence of TMD and worse quality of life based on the axis I of the RDC/TMD. In addition, there was a direct relationship between a greater duration and intensity of TMD pain and worse quality of life [4–6, 14–22, 22, 23, 23, 24, 24, 25, 25, 26, 26, 27, 27, 28, 28, 29, 29, 30, 34].

The great majority of the articles selected in this systematic review used the RDC/TMD axis I as the diagnostic method for TMD [36]. However, the use of the RDC/TMD axis II has not been applied in several studies [4, 14–16, 19, 22–24, 27, 28, 31–35]. This is paramount when the objective is to evaluate quality of life, because the axis II is focused on the TMD related psychosocial aspects (i.e., somatization, anxiety, depression, and oral quality of life questions) and should be used in future studies [37]. In fact, in a literature review, it was pointed out that more than affecting the quality of life, pain also influences the social and psychological aspects of the patient, leading to anxiety, depression, and the intensification of existing pathologies [38]. Another study showed a high prevalence of depressive and anxiety symptoms in patients with chronic pain [39].

The OHIP versions 14 and 49 were used in the majority of the studies; which is important because it describes oral health-related quality of life variables. On the other hand, the WHOQOL and SF-36 are validated questionnaires that involve general health-related quality of life variables, which is important for TMD as a multidisciplinary condition [4]. In addition, the WHOQOL has a sub-division involving the environment and the individuals as a whole; and the SF-36 is mainly focused on the mental and psychological health of patients [40, 41]. Since TMD involves the individual systemically, future studies should use more general health-related quality of life questionnaires.

Based on the findings in this systematic review, it is possible to observe that all included studies showed some relationship between the presence of TMD and worse quality of life based on the axis I of the RDC/TMD. In addition, there was a direct relationship between a greater duration and intensity of TMD pain and worse quality of life [4–6, 14–22, 22, 23, 23, 24, 24, 25, 25, 26, 26, 27, 27, 28, 28, 29, 29, 30, 34].

In relation to the meta-analysis, only a few studies could be included due to the wide range of methodological variations, the lack of a clear exposure, and the absence of the necessary data in many studies. However, it was possible to observe that TMD negatively influenced the quality of life when compared to the non-TMD population, mainly for the individuals classified in groups I and III, with group II having the least impact on the quality of life but with a significant statistical difference. This factor can be explained mainly by worse pain levels in groups I and III as compared to group II, as pointed out in many TMD studies as well as in a chronic musculoskeletal pain systematic review [4, 6, 14–20, 24–27, 33, 34, 42]. In addition, the presence of depression and somatization, reported worse in group I as compared to II [43], also negatively impacted the patients' quality of life [6, 15, 17, 20, 25]. Other study found a positive correlation between pain severity and both anxiety and depression symptoms, suggesting that the therapeutic intervention for anxiety and depression symptoms can be even more necessary in patients with more severe pain [44].

These quality of life differences could be attributed to the role of gender; considering that women present two times greater the risk of developing TMD, seeking more treatment in general and perceiving more pain [45, 46]. However, the literature has been contradictory regarding the role of gender difference in the severity and prevalence of quality of life [20, 22, 24], and only one study found worse functional limitation in women [24].

Due to the high heterogeneity found here, it was necessary to use the random effect analysis, where the observed effect is an estimate of its real effect and follows a general distribution, with smaller studies gaining greater weight as compared to studies with larger sample sizes [47, 48]. In order to improve future meta-analyses and to reduce heterogeneity and biases, we suggest that future studies apply either the RDC/TMD or DC/TMD axes I and II and standard quality of life measures in cases and controls, preferably from the general population, considering that only two studies in this review were population-based [16, 26]. In addition, they should also report the size of the sample, the median, the standard deviation, not only for the entire TMD sample, but also for the RDC/TMD or DC/TMD axis I groups I, II and III. The use of the RDC/TMD axis II is extremely important because this axis involves depression, somatization and pain intensity and disability, aspects that interfere in the TMD as shown in many studies [6, 15, 17, 20, 25]. Finally, the sample source, the method of blindness, the number and qualification of examiners, and the inclusion and exclusion criteria in both cases and controls should be provided to avoid undiagnosed TMD patients among controls [49, 50].

Regarding publication biases, it was possible to observe a bias towards positive results in Fig. 3a–d. This fact can be explained, because the included studies evaluated the quality of life in TMD patients and all demonstrated that the quality of life was worse in all TMD groups, but with different severities according to TMD groups with higher pain intensity and disability. Some studies have shown a direct relationship between both the duration of pain and pain intensity with poor quality of life in patients with TMD, showing that pain caused by TMD is one of the main reasons for the quality of life scores [6, 14–17]. Coherently, most articles clearly indicated that groups I and III, which have worse pain intensity, have worse quality of life as compared to group II of the RDC/TMD axis I [5, 18–27]. According to our results also, RDC/TMD axis I groups with higher pain levels (i.e., groups I and III) had worse quality of life as compared to the one with lower pain levels (i.e., group II).

Future studies should use not only the RDC/TMD or DC/TMD axis I to assess signs and symptoms of TMD, but also axis II in order to evaluate how quality of life-related variables (i.e., somatization, anxiety, depression, oral quality of life, and pain disability) are affected by TMD [37–39]. Future studies should also used standard general health-related quality of life questionnaires, and not only oral related quality of life ones, due to the TMD multidisciplinary etiology and management [4]. It is important that future investigations use the RDC/TMD or DC/TMD to select cases and controls in order to prevent contamination in both groups, preferably drawn from the general population with larger samples [16, 26]. Additionally, it should be assessed also if the TMD-related quality of life differences observed could be attributed to the role of gender [20–24]. Finally, complete data collection methodology of TMD and quality of life should be reported for all TMD diagnostic groups (i.e., muscle or TMJ related disorders) [49, 50].

In summary, this systematic review and meta-analysis has shown that quality of life is directly related to the pain intensity and disability reported by TMD patients. Therefore, TMD conditions where patients report less pain (i.e., disc displacements) have less impact in their quality of life than those with more pain (i.e., muscle disorders or arthralgia/arthritis/arthrosis).

## Conclusions

It can be concluded based on the results of this systematic review and meta-analysis that TMD patients have worse quality of life, which is directly related to higher pain intensity and disability reported by patients in the RDC/TMD groups I and III (i.e., muscle disorders and arthralgia/arthritis/arthrosis, respectively) as compared to group II (i.e., disc displacements). Future investigations should include general-health related quality of life questionnaires, provide complete data and data methodology in all TMD diagnostic groups, and use TMD validated diagnostic methods in order to select both TMD cases and controls.

## Data Availability

Data is available on request.
